# Protocol of the Study on Emergency Health Care Workers’ Responses Evaluated by Karasek Questionnaire: The SEEK-Study Protocol

**DOI:** 10.3390/ijerph18084068

**Published:** 2021-04-12

**Authors:** Jean-Baptiste Bouillon-Minois, Marion Trousselard, Bruno Pereira, Jeannot Schmidt, Maelys Clinchamps, David Thivel, Ukadike Chris Ugbolue, Farès Moustafa, Céline Occelli, Guillaume Vallet, Frédéric Dutheil

**Affiliations:** 1 Emergency Department, CHU Clermont-Ferrand, 63000 Clermont-Ferrand, France; jschmidt@chu-clermontferrand.fr (J.S.); fmoustafa@chu-clermontferrand.fr (F.M.); 2 LaPSCo, Université Clermont Auvergne, CNRS, 63000 Clermont-Ferrand, France; guillaume.vallet@uca.fr (G.V.); fdutheil@chu-clermontferrand.fr (F.D.); 3 French Armed Forces Biomedical Research Institute, BP73, 91223 Brétigny-sur-Orge, France; marion.trousselard@gmail.com; 4 APEMAC/EPSAM, EA 4360, Ile du Saulcy, BP 30309, 57006 Metz, France; 5 Clinical Research and Innovation Direction, CHU Clermont-Ferrand, 63000 Clermont-Ferrand, France; bpereira@chu-clermontferrand.fr; 6 Occupational and Environmental Medicine, CHU Clermont-Ferrand, 63000 Clermont-Ferrand, France; maelys.clinchamps@gmail.com; 7 Laboratory of the Metabolic Adaptations to Exercise under Physiological and Pathological Conditions (AME2P), Université Clermont Auvergne, 63000 Clermont-Ferrand, France; david.thivel@uca.fr; 8 Institute for Clinical Exercise & Health Science, University of the West of Scotland, Glasgow G720LH, UK; U.Ugbolue@uws.ac.uk; 9 Department of Biomedical Engineering, University of Strathclyde, Glasgow G1 1XQ, UK; 10 Emergency Department, CHU Nice, 06000 Nice, France; occelli.c@chu-nice.fr; 11 Emergency Department, Université Côte d’Azur, 06000 Nice, France

**Keywords:** Karasek questionnaire, job demand, job strain, shiftwork, nutrition, emergency physician, stress, job control, biomarkers

## Abstract

Background: Stress is a significant public health concern that can be self-evaluated using the job control demands model from Karasek. Emergency health care workers are particularly exposed to stress because of the intrinsic characteristics associated with the job (i.e., life-threatening emergencies, overcrowding, lack of bed spaces). However, these attributes have never been studied using the Karasek model. Methods: An observational, prospective, multicentric study in French Emergency Departments will be conducted using a cohort of emergency health care workers. Four questionnaires before a control day and after a nightshift will be assessed every 5 years in the same emergency departments. Also, the Karasek questionnaire, a sociodemographic questionnaire, the Maslach Burnout Inventory scale, the Hospital Anxiety, Depression Scale, and a food intake questionnaire will be evaluated. Salivary biomarkers (cortisol, immunoglobulin A, lysozyme) will be collected from every emergency health care worker who consents to participating in the study. Conclusion: This study will provide a point of care for the emergency health care workers’ stress situation every 5 years. Ethics: This protocol was registered in Clinical Trials under the identification NCT02401607 after the French Ethics Committee’s approval.

## 1. Background

Chronic stress at work is a significant public health problem increasing morbidity and mortality [1]. Many employees complain about “stress” at work. In the medical field, burnout prevalence near or exceeding 50% has been documented in national studies conducted in both physicians in training and practicing physicians [2]. This is especially true in the Emergency Departments. Emergency health care workers are a particularly at-risk population. Indeed, their work is a complex interaction between stress due to life-threatening emergencies; overcrowding of the Emergency Department; lack of sleep; bad food repartition before, during, and after shifts; and accumulated fatigue [3]. Health care workers are considered as high-risk group for death by suicide [4]. Although men a higher risk of suicide in the general population, female physicians have higher suicide rates than men [5]. Specialties with a heavy workload, long shifts, and unpredictable hours (associated with sleep deprivation); stress-related situations (life and death emergencies) [6]; and easy access to a means of committing suicide [7] are particularly at risk of suicide [4,8,9]. Emergency health care workers are shift workers with scheduling irregularities regarding hours, duration, and holidays. Shift workers have bad well-being [10] and cardiometabolic risk factors (higher blood pressure [11], higher levels of triglycerides [12], higher risk of obesity [13], higher risk of heart disease [14], and higher metabolic syndrome [15]). Even if quantitative energy intake is the same between nightshift workers and day workers [16], qualitative food choices and habits are different [16]. Shift workers consume more snacks, alcohol, and confectioneries and eat more frequently during the night [17], which possibly hurts circadian regulation [18]. This dysregulation is mainly impacted by the inappropriate secretion of insulin, ghrelin, and leptin [19], leading to diabetes [20]. To our knowledge, no study has evaluated the food intake of emergency physicians before nightshift, during nightshift, and after nightshift.

One way of measuring stress at work is the use of a self-reported psychological questionnaire. Among them, the job control demand model from Karasek is widely used to evaluate psychosocial factors at work [21,22]. It defines job demands and job control as the two broad work-related characteristics present in the environment of most occupations that can be incredibly stressful. Job demands refer to the psychological needs imposed by daily working activities such as mental workload, organizational constraints, and various types of conflicts (i.e., of the role, demands). Job control refers to the lack of decision latitude, specifically the freedom workers can have in realizing their job. Job control is composed of two components: Skill discretion (possibilities to use one’s skills) and decision authority (chances of making decisions that can reduce adverse effects of high psychological demands). Workers can perceive both job demand and job control as being low or high. The combination of high job demands and low job control results is called ‘job strain.’ This situation is the most aversive possible combination. Workers in the ‘job strain’ configuration are particularly at risk of low well-being [23], burnout [24], and ill-health [25], such as cardiovascular diseases [26,27].

Moreover, the Karasek job strain model is an independent predictor of acute coronary events [28]. Conversely, a combination of low demand and high control results in ‘low strain.’ The authors proposed an extension of the model in the 1980s to include another dimension: Worksite social support [29]. Support from colleagues and/or from the hierarchy acts as a buffer against complex combinations of job control and demands.

The Karasek job strain model has been assessed in 25,000 workers in the French SUMER study and classified as the main types of occupations [30]. However, no data regarding emergency health care workers’ scores at the Karasek questionnaire are available, even though burnout exposure is a well-known problem among health care workers [31,32]. This absence is regrettable as emergency health care workers often need to deal with life-threatening emergencies in a short amount of time, regularly following long shifts [33]. Karasek’s questionnaire is supposed to assess a long-term stable perception of work, despite the absence of a study comparing Karasek scores within different conditions in the same participant. It is also essential to explore protective factors that might help alleviate perceived stress and are relatively easy to integrate into medical organizations or daily lifestyle [34].

Regarding biological indicators of stress, several biomarkers of stress have been proposed [33,35,36]. To our knowledge, the relationships between biomarkers of stress and the Karasek job strain model have never been investigated. 

We are also interested in finding a precise way to evaluate work stress. Studies have shown contradictory results on biological markers of stress. The present study will use multiple stress markers, allowing for improved and more comprehensive results pertaining to how psychological stress interacts with biological variables.

Cortisol is the historical marker of stress. Nevertheless, several studies have not found modifications in cortisol levels. Thus, no correlations have been demonstrated between secretions of salivary cortisol and stress questionnaires among neonatal health care professionals [37]. Other studies have reported some underlying effects. For example, teaching, a profession with high levels of job stress and burnout, is associated with a significantly higher level of salivary cortisol during workdays versus free days [38].

Among the different biomarkers, our team previously highlighted the potential of salivary dehydroepiandrosterone (DHEA) to assess stress due to its stability (steroid) and its long half-life of 16 h [39]. Related to cortisol, some results have suggested that salivary DHEA levels are modified under stress [40]. Levels of salivary DHEA are also increased in anxiety symptoms [41]. The salivary DHEA’s half-life is about 15 h, making it a relatively stable concentration throughout the day, unlike cortisol. Therefore, salivary DHEA is an interesting marker.

Few studies have examined the response of salivary IgA in the case of work-related stress [42]. A recent study described the opposite relationship between stress and salivary IgA levels among healthy volunteers after a psychological stressor [43]. This marker’s advantages are its ease of access (saliva) and relatively long half-life (10–15 days), which makes it relatively stable.

Salivary lysozyme has been rarely studied. It does not appear to be modified after acute physical stress on healthy men [44], but studies have found but a negative correlation in university students during their final license exam [45] and in 50 male workers on an assembly line in China [46].

Most biological parameters mentioned here are related to personality questionnaires (locus of control, self-esteem) or perceived health (depression, quality of life, psychosomatic complaints). This will allow us to correlate participants’ perceptions of biological modification.

We performed a pilot study with 19 emergency physicians at the Clermont-Ferrand University Hospital. The Karasek questionnaire interview results showed a low decision latitude (59.67 ± 6.44; score below 71) and a high psychological demand (29.39 ± 4.07; score below 20) in this population, illustrating the definition of a stressful work situation (high workload with low autonomy). This state was fortunately balanced by a critical perceived social support (29.94 ± 3.21; score greater than 24). The results demonstrate a perfect illustration of inadequacy between professional demands (taking care of patients regardless of their numbers, varying according to hours and days) and organizational constraints (lack of beds spaces, for example, a situation where decision latitude is nonexistent, generating stress). Our pilot study permits us to situate the emergency physicians on the SUMER study, which lists the mean scores of psychological demands and decision latitude by professional family. This comparison highlights emergency physicians’ extraordinary situation, with decision latitude among the lowest and the highest psychological needs. Thus, it becomes interesting to appraise stress in this population through seeking solutions to reduce burnout.

Therefore, the objective of this manuscript was to present the design of a study protocol to examine well-being in emergency health care workers (the SEEK-study). The acronym SEEK is “Study on Emergency physicians’ responses Evaluated by Karasek questionnaire”. The primary objective is to assess and determine Karasek scores in a large sample size of emergency health care workers. The secondary objectives is to evaluate whether there is a change in work perception both in the short term (on leave and after a nightshift) and long term (with the reproducibility of this study every 5 years). Secondly, we will evaluate the food intake before, during, and after the nightshift and explore its relationship with Karasek’s score and stress biomarkers. We will further explore Karasek’s associations with some biomarkers of stress and protective factors.

## 2. Materials and Methods

### 2.1. Study Design

**Figure 1:**
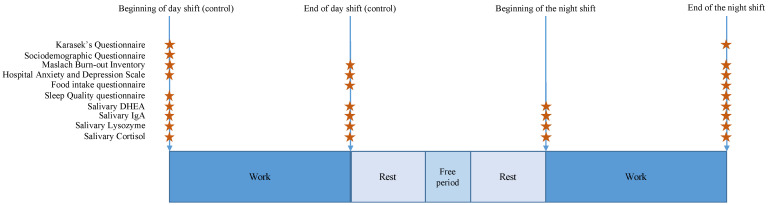
The Study on Emergency physicians’ responses Evaluated by Karasek questionnaire (SEEK) protocol.

Our study will be an observational and prospective study. It will be a multicenter, French study based on the observation of healthy volunteers without invasive sampling in France. We will record different variables and data from the study population without interventions. The study design is described in Figure 1. We will provide repeated measures in the same departments (each measurement time comprising of 24 h, including a nightshift, and 24 h, including a dayshift). We will create a cohort of workers that will be followed every 5 years if they are still working in the Emergency Department. However, we can include new health care workers if needed.

### 2.2. Study Settings

According to our pilot study, we evaluate the time required by the participant at about 15 min per phase.

For the first phase, investigators will assess the Karasek’s questionnaire, food intake questionnaire, Maslach Burnout Inventory, and Hospital Anxiety Depression Scale (HAD) among Emergency health care workers at the beginning of the control day (around 8:30 am). A sample of saliva in an Eppendorf will be recovered at the same time. Saliva collection will be brought in a cooler to the Institute of Occupational Medicine of Clermont-Ferrand by the end of the day. The samples will be stored at −80 °C. The experimenter will also deliver a pre-stamped envelope for the return of 2 Eppendorf tubes (saliva collection) and questionnaires at the end of the day.

Considering the difficulties of obtaining homogenous planning with all emergency providers, the time passed between the first and the second phase will not be the same between all providers. However, the 2 phases need to be separated by at least 1 week.

For the second phase, investigators will assess the same questionnaires with the saliva collection at the beginning and the end of a 24-hour or 14-hour nightwork session (up to 1 week later). This will permit the evaluation of the relationships between modifications in questionnaire answers and biological responses by comparing results during a control day versus results at the end of a work session, including a nightshift.

The third phase of the SEEK protocol is a longitudinal follow-up. This study design will be reproduced every 5 years in all Emergency Departments. If possible, i.e., if they are already in the same Emergency Department, we will follow-up on the initial cohort. We choose a follow-up of 5 years because it corresponds to the residency program duration in Emergency Medicine in France. We will program 4 sessions for a total of 20 years.

### 2.3. Ethics and Dissemination

A French Ethics committee (Comité de Protection de Personnes Sud-Est I, CHU Saint-Etienne) approved this study protocol on 3 November 2014, with reference DC-2014-2151. This protocol was registered in ClinicalTrials under the identification NCT02401607.

The results of questionnaires will be coded and managed through ReDCap© software with pseudonymous codification.

### 2.4. Eligibility Criteria

To fulfill the inclusion criteria, every health care worker will need to over 18 years of age and working in an Emergency Department in France. No requirement of experience is necessary. Healthcare workers will be excluded for refusal of participation, intercurrent pathology with hormones disabilities, impracticability to take the follow-up, psychopathology with depression or anxiety, taking any drugs that modulate inflammation or hormones levels, and pregnancy.

### 2.5. Recruitment Process

During each part of the study, 120 emergency health care workers will be recruited as participants. During the recruitment phase, all emergency health care workers will individually receive an information letter attached by email. Acceptance or refusal will be given by mail to avoid any subordination effect between the experimenter and the recruited. If they agree, they will have 8 days to sign the consent form to participate in the study. For privacy purposes, the question about their medical history will not be asked by email. Instead, a list of non-inclusion criteria will be given in the information letter. Thus, if any exclusion criteria are present, they can refuse participation without any need for further explanation.

### 2.6. Outcomes

The primary outcome is the participants’ score with respect to the Karasek questionnaire in terms of the 3 dimensions, namely psychological demands, decision latitude, and social support at work.

Secondary outcomes are different biological measures of stress (cortisol concentrations, DHEA-S, salivary IgA, and lysozymes); some psychological questionnaires (see below for details), namely the Maslach Burnout Inventory, the Hospital Anxiety and Depression Scale; and some question about stress (at home, in life, and at work), about sleep quality, and about mental and physical fatigue. Lastly, we will assess food intake using a quantitative and qualitative questionnaire.

## 3. Results

### 3.1. Justification of the Sample Size

The number of subjects required justification is based on the ability to recruit participants, namely 120 subjects. Previous studies have shown an acceptance rate above 80% in the emergency health care workers’ population; power estimates will be provided based on 100 subjects included. In addition to the expected variability, this type of study can have a fixed margin of error on the estimated sample size (reliability of the sample). Referring to the data of our pilot study, the Karasek questionnaire found low decision latitude (59.67 ± 6.44, 6% higher than 71), a significant psychological burden (29.39 ± 4.07; 94% greater than 20), and high social support (29.94 ± 3.21, 100% above 24), meaning that with at least 100 subjects considered, the error margin will be very satisfying that is respectively ±1.25, ±0.8 and ±0.63. If we consider the different dimensions score as categorical (<> 71 <> 20 and <> 24), accuracy will be ±5% with n = 100.

Furthermore, simulations on secondary objectives can complete this justification. To see a change in the emergency health care workers’ work perception during a control day versus the results at the end of a nightshift, 100 subjects will show minimum differences of the order of 2.35, 1.48, and 1.17 for decision latitude dimensions, psychological demand, and social support, with a type 1 error risk of 5%, a power of 95% and a correlation coefficient of 0.5 (matched condition).

With 100 subjects, and given the assumptions described above, 5 predictors can be explored to identify factors that explain emergency work perception for a power greater than 80% [47].

### 3.2. Data Collection and Management

Seven questionnaires will be presented to every participant. All psychological assessments will be self-reported.

#### 3.2.1. Karasek Questionnaire

Karasek’s questionnaire [21] was French-validated by Brisson et al. in 1998 [48]. The questionnaire includes 26 items measuring the 2 burnout sources: The decision latitude, with 9 items, and psychological job demands, with 9 items. For each item, the subject is asked to respond using a 4-level Likert-type scale. Thus, a high score in the first dimension means that the worker acts autonomously and is less exposed to burnout. Meanwhile, a high score in the second dimension implies that the subject is under a heavy workload, leading to severe psychological stress. Social support, explored by 8 items, acts as a burnout modulator: High social support helps to better tolerate situations linked with burnout emergence. The analysis is based on the median of each factor.

The psychological demand is calculated with the following formula: Q10 + Q11 + Q12 + (5 − Q13) + Q14 + Q15 + Q16 + Q17 + Q18. A score below 20 reflects a low psychological demand. The decision latitude is calculated with the following formula: 4 × Q4 + 4 × (5 − Q6) + 4 × Q8 + 2 × (5 − Q2) + 2 × Q5 + 2 × Q7 +2 × Q1 + 2 × Q3 + 2 × Q9. A score below 71 reflects low decision latitude. Finally, the social support is calculated with the following formula: Q19 + Q20 + Q21 + Q22 + Q23 + Q24 + Q25 + Q26. A score below 24 reflects low social support [22]. In a previous study performed by our team, the Cronbach’s alphas for job demands, job control, and social support were 0.58, 0.99, and 0.99, respectively [49].

In summary, the Karasek questionnaire postulates that a work situation generates stress if it combines high psychological demands, low decision latitude, and low social support from the work team or hierarchy. By combining autonomy and demand, 4 broad categories are defined:

− Relaxed work: Low demand, high autonomy
− Passive work: Low demand, low autonomy
− Active work: High demand, high autonomy
− Stressed, tense work: High demand, low autonomy

#### 3.2.2. Maslach Burnout Inventory (MBI) Scale

The MBI scale [50] evaluates 3 different components, giving a burnout evaluation for each component: Emotional exhaustion, depersonalization, and personal accomplishment. Dion and Tessier made a French-translated version of the MBI scale [51]. The scale is composed of 22 items measuring 3 different burnout dimensions: Emotional exhaustion, depersonalization, and personal accomplishment. For each item, the subject is asked to indicate using a 7-point scale the occurrence frequency of the feeling corresponding to a state. Thus, a high score in the first 2 subscales reveals a burnout state. In contrast, a high subscale score on achievement means that the subject feels fulfilled professionally (the achievement subscale is inverted). In a previous study performed by our team in a similar population, analysis of internal consistency yielded a Cronbach’s alpha of 0.785, indicating scale reliability [52].

#### 3.2.3. Hospital Anxiety and Depression Scale (HAD) Scale

Zigmond & Snaith’s (1983) HAD Scale explores anxiety and depression [53]. It was translated into French by Lépine in 2000 [54]. The scale is composed of 7 items exploring anxiety symptoms, and 7 others exploring depressive symptoms. Each item is given on a 4 degrees-of-severity scale. Anxiety and depression score ranges from 0 to 21. The generally admitted pathological thresholds score is 8. In a previous study performed by our team in a similar population, the Cronbach’s alphas for the components of depression and anxiety were 0.82 and 0.79, respectively [49].

#### 3.2.4. Sociodemographic Report

A unique questionnaire will be used to collect sociodemographic data such as age, sex, weight, height, and marital status. Information about medical history, drugs, and pregnancy (if female) will be collected. A second part of the questionnaire will assess the physical activity (number of hours per week and intensity) and sleep of every health care worker (number of hours and quality assess by a visual analogic scale from worst to best). Others visual analogic scales will assess stress and mental and physical tiredness.

#### 3.2.5. Place of Work

Information about the Emergency Department and hospital such as type of hospital (university or not), number of patients every year, and type of patients (adult, kids, both) will be collected.

#### 3.2.6. Occupational Characteristics

These include seniority (within the hospital, within the Emergency Department, and as an emergency occupation), current position (physician, internship or externship student, nurses, caregivers, cleaners, secretary, hospital porter, other), and type of contract (permanent or temporary). For physicians, these characteristics also include the type of emergency diploma or initial specialty and more details on contracts (professors, senior lecturer, hospital practitioner, assistant).

#### 3.2.7. Food Intake

To study the qualitative and quantitative food intake of emergency health care workers, participants will be given a questionnaire. Three timepoints will be examined—before, during, and after nightshift—and compared to a control day. We will ask participants to complete a 3-day dietary recall that will be explained and detailed to them by a member of the investigation team. We will ask to participants to indicate all the details regarding the food ingested at each meal and in-between meals as precisely as possible. A specialized dietician will provide details in the diary as well as some guidance on how to complete it. Subsequently, the diaries will be reviewed with the participants and the dietitian during a 45-min interview [55]. All data will be pooled and translated in kcal., and the quantity of lipids, carbohydrates, and proteins will be evaluated using the software Nutrilog^®^.

#### 3.2.8. Biological Samples

Several assays will be performed on the saliva sample. Biological analyses will be conducted by the Institute of Occupational Medicine of the Medical School of Clermont-Ferrand. We will measure salivary cortisol concentrations, DHEA-S, sIgA, and lysozymes. These parameters have been reported in the literature as correlated with the level of stress or workload. Only steroids (cortisol and DHEAS) with good stability will be measured in both samples. Peptides (sIgA and lysozyme) will be measured only during the dayshift (control). The samples made after the work session will be sent by post, which does not guarantee sIgA and lysozyme preservation.

### 3.3. Data Analysis

Continuous variables will be presented as mean and standard-deviation or median and interquartile range according to the statistical distribution. The normality assumption will be assessed using Shapiro–Wilk test. Categorical variables will be expressed as numbers and associate percentage. The primary analysis will be mainly descriptive and will define the perception scores of emergency physicians work according to the Karasek questionnaire for each dimension composing the score (decision latitude, psychological demands, and social support), as quantitative and categorical variable. A correlation matrix analysis on quantitative variables (i.e., Karasek questionnaire and other psychological questionnaires) will be conducted using Pearson or Spearman correlation coefficients, in addition to principal component analysis (PCA). The Karasek questionnaire score’s evolution, obtained for each dimension, will be tested with a paired two-sample Student’s test, or nonparametric Wilcoxon test if necessary. Linear regression models and random-effects models will complete these analyses. These models will permit, with multivariable analyses and covariates’ introduction, to seek possible explanatory factors of the Karasek score (a dayshift (control) versus the end of 24 h or shift) and its evolution. The same statistical analysis plan will be used for continuous parameters collected after the dayshift (control) and after the 24-h shift (e.g., all biological samples). For categorical variables, Stuart-Maxwell test will be used. The comparisons for quantitative parameters (e.g., biological data, questionnaires’ scores) will be based on Student’s *t*-test or nonparametric Mann–Whitney test. The comparisons involving categorical parameters (gender, seniority) will be made with the Chi-squared test or Fisher’s exact test. All statistical analyses will be performed as two-sided tests, with a *p* < 0.05 considered as significant. Statistical analyses will be performed using the Stata software (version 16, StataCorp, College Station, TX, USA).

## 4. Discussion

This protocol seems particularly innovative considering the lack of emergency health care workers in the SUMER study. Indeed, those workers are mainly at risk of burnout [4]. Furthermore, and even if it was not the main objective, the recent actuality with the COVID-19 pandemic showed an increase in the workload for emergency health care workers. Indeed, even in the area with low incidence of first wave of COVID [56], overcrowding is increasing in the Emergency Departments [57]. Furthermore, the 20 years of follow-up will make it possible to assess the impact of local (an increase of the number of workers during shifts, change in the food quality and quantity…) and national policy (no more 24-h shifts, security rest after a nightshift) on the well-being of workers. This study will bring important data about emergency health care workers’ work perception and stress. No data on the Karasek questionnaire score currently enable u, to compare Emergency Medicine with other medical specialties. Indeed, the SUMER study did not include any emergency health care workers. The SUMER study [30] conducted in France on 25,000 employees in 2002–2003 found that all confounded professions produced average psychological demand scores of 21.5 for men and 21.8 for women, and average decision latitude scores of 71.9 for men and 68.8 for women. The distribution of these four broad categories was Relaxed 29.2%, Passive 25.2%, Active 25.2%, and Tense 20.4%. The SUMER study can be used to identify the psychological demands and decision latitude mean scores by professional family.

### Potential Limitations

The psychological questionnaires in this study are self-assessed. Even if those questionnaires are made to avoid bias, it is still possible that responses suffer from variation. As an observational study, we cannot control the 24 h work schedule. Some individuals’ sessions might be more demanding than others, thus bringing more important modifications to a biological sample.

## 5. Conclusions

This project will contribute to an overview of emergency health care workers’ conditions. The omission of their place in the SUMER study after the Karasek questionnaire will be corrected. Second, the outcome from this study, repeated every 5 years over 20 years, will provide an insight into whether or not improvements have occurred in the emergency health care working conditions and, if so, how this outcome will influence or impact health care policy. Last, the secondary outcomes will probably give our team the impetus to improve life quality by increasing quality and quantity of food intake or by the impact of new Emergency Department organization between two measures.

## Data Availability

No new data were created or analyzed in this study. Data sharing is not applicable to this article.
